# Lipoprotein(a) is a Prevalent yet Vastly Underrecognized Risk Factor for Cardiovascular Disease

**Published:** 2024-03-12

**Authors:** Jacob Rendler, Mia Murphy, Calvin Yeang

**Affiliations:** Division of Cardiology, Department of Medicine, UC San Diego, La Jolla, California, United States of America

## DESCRIPTION

**Figure 1:**
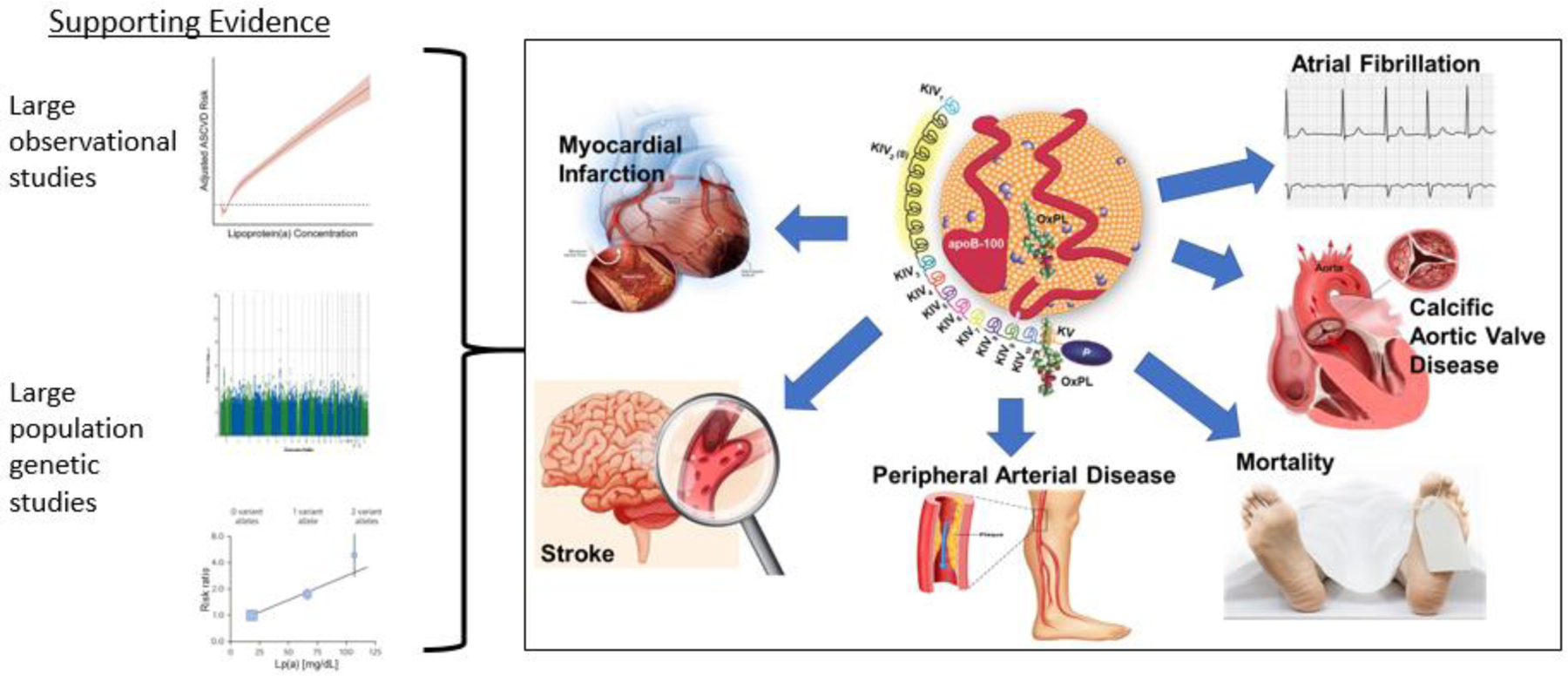
Multiple, large and high quality prospective, genome wide association, and mendelian randomization population studies have provided support for the causal role of Lp(a) in various facets of cardiovascular disease.

Lipoprotein(a) (Lp(a)), consisting of an apoB containing lipoprotein with similarities to low density lipoprotein (LDL) covalently bound to apolipoprotein(a) [apo(a)], is a common cardiovascular disease (CVD) risk factor with elevated levels present in 10–20% of the population [1–3]. The composition of Lp(a) has informed the mechanisms by which it mediates CVD. Lp(a) is the major lipoprotein carrier of pro-inflammatory and pro-calcific oxidized phospholipids, shares pro-atherogenic features with other cholesterol rich apoB lipoproteins, and additionally may be pro-thrombotic [4,5]. An established and expanding body of high quality epidemiological and human genetic evidence base supports a causal role of Lp(a) as a risk factor for CVD including coronary and peripheral arterial disease and ischemic stroke, calcific aortic valve disease, and atrial fibrillation (Figure 1), and has reviewed in recent scientific statements by the American Heart Association (AHA) and European Atherosclerosis Society (EAS) [6,7].

Knowledge of Lp(a) levels informs comprehensive CVD risk assessment and international medical societies including the AHA, European Society of Cardiology (ESC), National Lipid Association (NLA), American Association of Clinical Endocrinology (AACE), Canadian Cardiovascular Society (CCS), and EAS have provided guidance on Lp(a) testing (Table 1). There is consensus between these organizations supporting Lp(a) testing in individuals with a family or personal history of premature CVD. Moreover, the ESC, EAS, and CCS recommend Lp(a) screening in all adult individuals to identify those with elevated levels contributing to higher CVD risk. As Lp(a) levels are ~ 90% genetically determined with only modest potential fluctuations influenced by age, lifestyle, and co-morbidities, a single test would be sufficient to identify or exclude individuals with elevated Lp(a) levels >50 mg/dL (125 nmol/L) associated with increased CVD risk [6,7].

**Figure 2:**
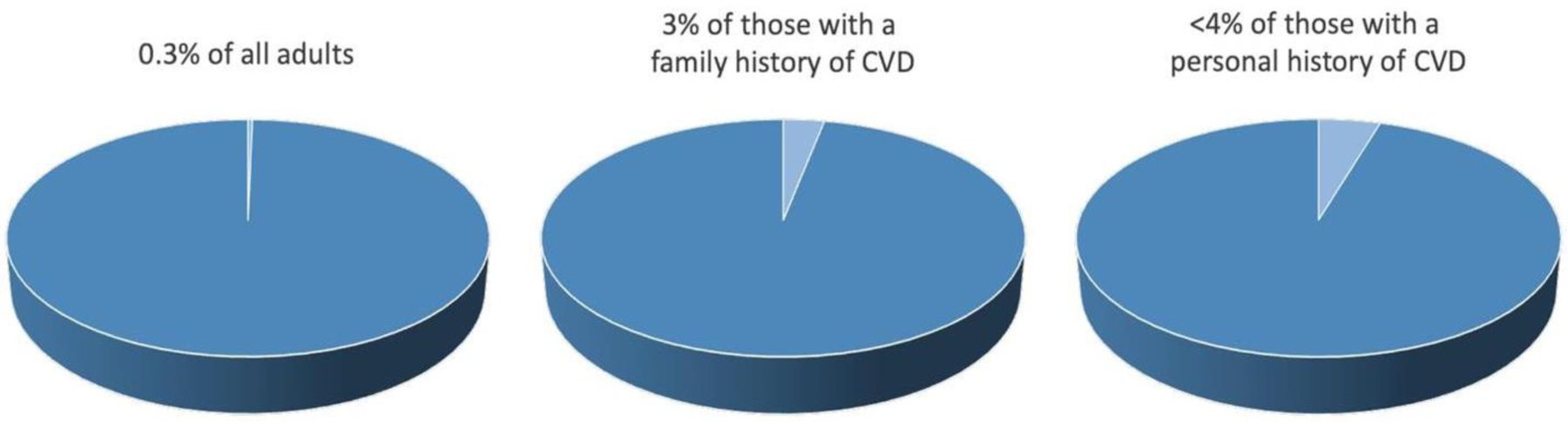
Prevalence of Lp(a) utilization in all adults or individuals with a family or personal history of cardiovascular disease in an academic health system consisting of >5.5 million persons [12]. Note: 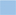 / Lp(a) testing; 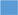 / No Lp(a) testing.

Despite a 10%−20% prevalence of elevated Lp(a), utilization of Lp(a) testing in clinical practice has been long felt to be poor. Recently, Lp(a) testing rates have been systematically quantified confirming prior anecdotal suspicions. A study consisting of >5.5 million adults across 6 academic health systems in California from 2013–2021 revealed only 0.3% of the population had Lp(a) testing (Figure 2) [8]. Of the patients that had been screened for CVD risk factors, identified by having at least one lipid panel, just 1.8% also had Lp(a) testing. Only less than 4% of individuals with either a personal or family history of CVD had Lp(a) testing. Similarly low rates of Lp(a) utilization were reported in other health systems across the US, with 0.06%−3% of individuals tested [9,10]. Moreover, gender and racial disparities in Lp(a) testing rates exist–more likely in men compared to women and whites compared to blacks [8].

Why is Lp(a) so infrequently tested despite being one of the most common CVD risk factors? There have been several reasons suggested anecdotally. These include an underrecognition of the evidence base supporting Lp(a) as a causal CVD risk factor and/or guideline indications for Lp(a) testing. Clinicians may not be fully aware of existing guidance on how to incorporate Lp(a) into CVD risk assessment and modification for their patients and instead operate with the assumption that Lp(a) testing would not change clinical management. While CVD outcomes trials with specific and potent Lp(a) lowering therapies are ongoing, some clinicians may feel that readout of these trials are required before changing practice patterns around Lp(a) testing. Additionally, clinical laboratories vary in their choice of calibrators for Lp(a) with some reporting values in mg/dL and others in nmol/L. The lack of standardization for Lp(a) measurements may be a barrier for testing due to confusion with interpretation or perceived imprecision of currently available assays. Lastly, concerns around testing cost to the patient, insurance, and healthy system may influence Lp(a) utilization. The prevalence of these barriers, and their order of importance to clinicians will need to be objectively and systematically evaluated to fully understand and lead to action to improve Lp(a) utilization.

It is important to note that many, if not all of these potential barriers to Lp(a) testing can be overcome by more awareness of existing knowledge. Dissemination of the existing guidance for Lp(a) testing as well as risk assessment and modification for patients with elevated Lp(a) at local and national continuing medical education (CME) events attended by primary care physicians, cardiologists, vascular specialists, lipid specialists, and stroke neurologists is one opportunity to empower clinicians to check Lp(a) when appropriate. Multiple ongoing clinical trials evaluating targeted therapies that potently lower Lp(a) by 25%−100% (Table 2), fuel potential that additional risk modifying options may be soon available for patients with elevated Lp(a). However, recognition of patients with elevated Lp(a) through testing is potential for enrolling adequately powered trials and the confidence in interpreting their results. Regarding Lp(a) assays, efforts to standardize Lp(a) measurements in nmol/L are ongoing [11], however, routinely available clinical assays are sufficient for identification of patients with elevated Lp(a) levels–either >50 mg/dL or >125 nmol/L. Lastly, Lp(a) testing is relatively inexpensive and cost effective. Lp(a) tests are simple immunoassays with cost to insurance or patients ranging from $21-$99 (Table 3). As Lp(a) levels are predominately genetically determined, a single Lp(a) test would sufficiently determine Lp(a) attributable risk in most individuals.

## CONCLUSION

Lp(a) testing is essential for comprehensive CVD risk evaluation and is indicated in those with a personal or family history of premature CVD, if not all individuals. There is an urgent need to systematically understand the barriers around Lp(a) utilization. A survey based assessment of awareness, beliefs and practice patterns with Lp(a) testing broadly amongst primary care clinicians, cardiologists, lipidologists, stroke neurologists, vascular specialist, population health, and payors is now needed to guide practice changing education and implementation to improve Lp(a) testing.

## Figures and Tables

**Table 1:** Recommendations for Lp(a) testing by major international clinical guidelines and expert consensus statements. American Heart Association (AHA)/American College of Cardiology (ACC) [8], European Society of Cardiology (ESC) [9], National Lipid Association (NLA) [10], American Association of Clinical Endocrinology (AACE) [1,1], Canadian Cardiovascular Society (CCS) [12], European Atherosclerosis Society (EAS) [7].

	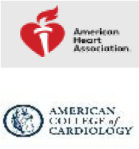	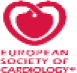	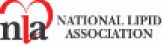	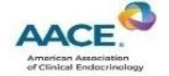	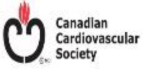	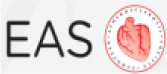
	2018	2019	2019	2020	2021	2022
All individuals to identify those at high CVD risk		X			X	X
Family/personal history of premature ASCVD	X	X	X	X	X	X
Specific Indications			To aid in decision making for statins in those with intermediate ASCVD risk	Individuals with south Asian or African ancestry, Individuals with a 10 year ASCVD risk ≥ 10%		Youth with a history of ischemic stroke and no other identifiable risk factors.
			To identify a possible cause for less than anticipated pharmacologic LDL-C lowering	Patients with statin resistance		
			To identify those at risk for progressive aortic stenosis	Patients with a personal or family history of aortic stenosis		

**Table 2:** Emerging targeted Lp(a) lowering therapies.

Therapy	Mechanism of action	Modification	% Lp(a) reduction	Current clinical trial stage
Muvalaplin	Inhibits the assembly of Lp(a)		^ ~^25–65% [17]	Phase 1
Pelacarsen	AntiSense Oligonucleotide (ASO)	N-acetyl galactosamine (GalNAc)	35–80% [18]	Phase 3
Olpasiran	Small interfering RNA (siRNA)	GalNAc	70–100% [19]	Phase 3
SLN360	siRNA	GalNAc	46–98% [20]	Phase 2
Lepodisiran	siRNA	GalNAc	41–97% [21]	Phase 2

**Table 3:** Lp(a) testing costs at select clinical laboratories.

Laboratory name	Lp(a) calibrator units	Cost
Labcorp	nmol/L	$49
Quest	nmol/L	$91
Boston heart diagnostics	nmol/L	$21
empowerDX	mg/dL	$99
Letsgetchecked	mg/dL	$89
Healthlabs	nmol/L	$49

## References

[1] Nordestgaard BG, Langsted A. Lipoprotein(a) as a cause of cardiovascular disease: Insights from epidemiology, genetics, and biology. J Lipid Res. 2016;57(11):1953–1975. 27677946 10.1194/jlr.R071233 PMC5087876

[2] Varvel S, McConnell JP, Tsimikas S. Prevalence of elevated Lp(a) mass levels and patient thresholds in 532 359 patients in the United States. Arterioscler Thromb Vasc Biol. 2016;36(11):2239–2245.. 27659098 10.1161/ATVBAHA.116.308011

[3] Patel AP, Wang M, Pirruccello JP, Ellinor PT, Ng K, Kathiresan S, Lp (a)(lipoprotein [a]) concentrations and incident atherosclerotic cardiovascular disease: New insights from a large national biobank. Arterioscler Thromb Vasc Biol. 2021;41(1):465–474. 33115266 10.1161/ATVBAHA.120.315291 PMC7769893

[4] Boffa MB, Koschinsky ML. Oxidized phospholipids as a unifying theory for lipoprotein(a) and cardiovascular disease. Nat Rev Cardiol. 2019;16(5):305–318. 30675027 10.1038/s41569-018-0153-2

[5] Yeang C, Wilkinson MJ, Tsimikas S. Lipoprotein(a) and oxidized phospholipids in calcific aortic valve stenosis. Curr. Opin. Cardiol. 2016;31(4):440–450. 27205885 10.1097/HCO.0000000000000300 PMC4956483

[6] Reyes-Soffer G, Ginsberg HN, Berglund L, Duell PB, Heffron SP, Kamstrup PR, Lipoprotein(a): A genetically determined, causal, and prevalent risk factor for atherosclerotic cardiovascular disease: A scientific statement from the American heart association. Arterioscler Thromb Vasc Biol. 2022;42(1):e48–60. 34647487 10.1161/ATV.0000000000000147 PMC9989949

[7] Kronenberg F, Mora S, Stroes ESG, Ference BA, Arsenault BJ, Berglund L, Lipoprotein(a) in atherosclerotic cardiovascular disease and aortic stenosis: A European Atherosclerosis Society consensus statement. Eur Heart J. 2022;43(39):3925–3946. 36036785 10.1093/eurheartj/ehac361 PMC9639807

[8] Bhatia HS, Hurst S, Desai P, Zhu W, Yeang C. Lipoprotein(a) testing trends in a large academic health system in the United States. J Am Heart Assoc. 2023;19;12(18):e031255. 37702041 10.1161/JAHA.123.031255 PMC10547299

[9] McGowan M, Wilemon K, Ahmed C, Myers K, MacDougall D. Characterization of lipoprotein(a) measurement in a large US healthcare dataset. J Clin Lipidol. 2022;16(3):e36–e37.

[10] Kelsey MD, Mulder H, Chiswell K, Lampron ZM, Nilles E, Kulinski JP, Contemporary patterns of lipoprotein(a) testing and associated clinical care and outcomes. Am J Prev Cardiol. 2023;14:100478. 37025553 10.1016/j.ajpc.2023.100478 PMC10070377

[11] Standardization for lipoprotein(a) Measurement in Humans | NHLBI, NIH. 2019.

[12] Grundy SM, Stone NJ, Bailey AL, Beam C, Birtcher KK, Blumenthal RS, 2018 AHA/ACC/AACVPR/AAPA/ABC/ACPM/ADA/AGS/APhA/ASPC/NLA/PCNA Guideline on the management of blood cholesterol: A report of the American college of cardiology/American Heart Association task force on clinical practice guidelines. Circulation. 2019;139(25):e1082–143. 30586774 10.1161/CIR.0000000000000625 PMC7403606

[13] Mach F, Baigent C, Catapano AL, Koskinas KC, Casula M, Badimon L, 2019 ESC/EAS Guidelines for the management of dyslipidaemias: Lipid modification to reduce cardiovascular risk: The task force for the management of dyslipidaemias of the European Society of Cardiology (ESC) and European Atherosclerosis Society (EAS). Eur Heart J. 2020;41(1):111–188. 31504418 10.1093/eurheartj/ehz455

[14] Wilson DP, Jacobson TA, Jones PH, Koschinsky ML, McNeal CJ, Nordestgaard BG, Use of lipoprotein(a) in clinical practice: A biomarker whose time has come. A scientific statement from the national lipid association. Don P. Wilson, MD, on behalf of the writing group. J Clin Lipidol. 2019;16(5):77–95 10.1016/j.jacl.2022.08.007 36068139

[15] Handelsman Y, Jellinger PS, Guerin CK, Bloomgarden ZT, Brinton EA, Budoff MJ, Consensus statement by the American association of clinical endocrinologists and American college of endocrinology on the management of dyslipidemia and prevention of cardiovascular disease algorithm-2020 executive summary. Endocr Pract 2020;26(10):1196–1224. 33471721 10.4158/CS-2020-0490

[16] Pearson GJ, Thanassoulis G, Anderson TJ, Barry AR, Couture P, Dayan N, Canadian cardiovascular society guidelines for the management of dyslipidemia for the prevention of cardiovascular disease in adults. Can J Cardiol. 2021;37(8):1129–1150. 33781847 10.1016/j.cjca.2021.03.016

[17] Nicholls SJ, Nissen SE, Fleming C, Urva S, Suico J, Berg PH, Muvalaplin, an oral small molecule inhibitor of lipoprotein(a) formation: A randomized clinical trial. J. Am. Med. Assoc. 2023;330(11):1042–1053. 10.1001/jama.2023.16503 PMC10463176 37638695

[18] Tsimikas S, Karwatowska-Prokopczuk E, Gouni-Berthold I, Tardif JC, Baum SJ, Steinhagen-Thiessen E, Lipoprotein(a) reduction in persons with cardiovascular disease. N Engl J Med. 2020;382(3):244–255. 31893580 10.1056/NEJMoa1905239

[19] O’Donoghue ML, Rosenson RS, Gencer B, López JA, Lepor NE, Baum SJ, Small interfering RNA to reduce lipoprotein(a) in cardiovascular disease. N Engl J Med. 2022;387(20):1855–1864. 36342163 10.1056/NEJMoa2211023

[20] Nissen SE, Wolski K, Balog C, Swerdlow DI, Scrimgeour AC, Rambaran C, Single ascending dose study of a short interfering RNA targeting lipoprotein(a) production in individuals with elevated plasma lipoprotein(a) levels. J. Am. Med. Assoc. 2022;327(17):1679–1687. 10.1001/jama.2022.5050 PMC8978050 35368052

[21] Nissen SE, Linnebjerg H, Shen X, Wolski K, Ma X, Lim S, Lepodisiran, an extended-duration short interfering RNA targeting lipoprotein(a): A randomized dose-ascending clinical trial. J. Am. Med. Assoc. 2023;330(21):2075–2083. 10.1001/jama.2023.21835 PMC10641766 37952254

